# Serum AP-endonuclease 1 (sAPE1) as novel biomarker for hepatocellular carcinoma

**DOI:** 10.18632/oncotarget.26555

**Published:** 2019-01-08

**Authors:** Devis Pascut, Caecilia Hapsari Ceriapuri Sukowati, Giulia Antoniali, Giovanna Mangiapane, Silvia Burra, Luca Giovanni Mascaretti, Matteo Rossano Buonocore, Lory Saveria Crocè, Claudio Tiribelli, Gianluca Tell

**Affiliations:** ^1^ Liver Research Center, Fondazione Italiana Fegato, ONLUS, AREA Science Park, Basovizza, Trieste, Italy; ^2^ Laboratory of Molecular Biology and DNA Repair, Department of Medicine (DAME), University of Udine, Udine, Italy; ^3^ Department of Medical Sciences, University of Trieste, Trieste, Italy; ^4^ Transfusion Medicine Department, Azienda Sanitaria Universitaria Integrata di Trieste (ASUITS), Trieste, Italy; ^5^ Clinica Patologie Fegato, Azienda Sanitaria Universitaria Integrata di Trieste (ASUITS), Trieste, Italy

**Keywords:** APE1, hepatocellular carcinoma, diagnosis, biomarker, DNA repair

## Abstract

Late diagnosis for Hepatocellular Carcinoma (HCC) remains one of the leading causes for the high mortality rate. The apurinic/apyrimidinic endonuclease 1 (APE1), an essential member of the base excision DNA repair (BER) pathway, contributes to cell response to oxidative stress and has other non-repair activities. In this study, we evaluate the role of serum APE1 (sAPE1) as a new diagnostic biomarker and we investigate the biological role for extracellular APE1 in HCC. sAPE1 level was quantified in 99 HCC patients, 50 non-HCC cirrhotic and 100 healthy controls. The expression level was significantly high in HCC (75.8 [67.3–87.9] pg/mL) compared to cirrhosis (29.8 [18.3–36.5] pg/mL] and controls (10.8 [7.5–13.2] pg/mL) (*p* < 0.001). The sAPE1 level corresponded with its protein expression in HCC tissue. sAPE1 had high diagnostic accuracy to differentiate HCC from cirrhotic (AUC = 0.87, sensitivity 88%, specificity 71%, cut-off of 36.3 pg/mL) and healthy subjects (AUC 0.98, sensibility 98% and specificity 83%, cut-off of 19.0 pg/mL). Recombinant APE1, exogenously added to JHH6 cells, significantly promotes IL-6 and IL-8 expression, suggesting a role of sAPE1 as a paracrine pro-inflammatory molecule, which may modulate the inflammatory status in cancer microenvironment. We described herein, for the first time to our knowledge, that sAPE1 might be considered as a promising diagnostic biomarker for HCC.

## INTRODUCTION

Hepatocellular carcinoma (HCC) ranks sixth as the most frequent cancer worldwide [1] with more than 800,000 new cases diagnosed every year. The late diagnosis still remains one of the leading causes for the high mortality rate [1]. The recent molecular characterization of the disease [2] provided new insights into the cellular networks involved in hepatocarcinogenesis becoming a source of potential new therapeutic targets as well as new biomarker to use in a clinical setting.

DNA repair pathways play a significant role in the cellular and organismal response to environmental exposure by maintaining the genome integrity and thus helping to prevent the onset of disease, aging phenotypes, and cancer development. Tumor cells can develop drug resistance *via* repair mechanisms that counteract the DNA damage from chemotherapy or radiation therapy, and DNA repair enzymes are emerging targets for novel anticancer strategies [3, 4].

The base excision DNA repair (BER) pathway handles simple alkylation and oxidative lesions arising from both endogenous and exogenous sources, including cancer therapy agents [5]. The essential BER enzyme, the apurinic/apyrimidinic endonuclease 1 (APE1), contributes to the regulation of oxidative stress responses and has other non-repair activities, such as regulating the expression of chemoresistance genes through direct and indirect mechanisms [6, 7]. By using APE1 knock-down models, we and others have demonstrated the pleiotropic ability of this protein to regulate the expression of hundreds of genes, involved in different biological processes, which are associated with cancer cell proliferation, invasion and drug resistance [8–11]. Interestingly, accumulating evidences indicate that APE1 may control gene expression via unsuspected functions in RNA metabolism [5, 12, 13] including miRNA expression [10], thus enhancing APE1 critical functions in tumor progression.

Well-known features linking APE1 and tumor development are its over-expression in many tumors and the correlation with the onset of chemoresistance in HCC and Non-Small Cell Lung Cancer (NSCLC), as well as neurologic, ovarian and breast tumors [11, 14]. Notably, its inhibition or down-regulation sensitizes cancer cells to DNA-damaging chemotherapeutic drugs and ionizing radiation [15]. Interestingly, a recent pioneering study found that plasmatic APE1 may represent a biomarker for predicting prognosis and therapeutic efficacy in NSCLC [16]. In fact, the chemotherapy-naïve serum APE1 level, which correlates with its tissue level, is inversely associated with progression-free survival of platinum-containing doublet chemotherapy; whereas post-treatment serum APE1 level is inversely associated with overall survival [16]. Interestingly, increasing evidences support the finding that extracellular APE1 secretion is a common feature of cancer cells, while the biological meaning remains to be elucidated but could be associated to triggering the inflammatory response of cancer microenvironment [17–19].

Despite several findings that associated APE1 to chemo- and radio resistance in tumors [16], marginal information exists in HCC. From our previous studies performed in cancer tissue biopsies, we defined a prognostic role for APE1 expression in HCC [20, 21]; however, nobody still evaluated the expression of APE1 in serum (sAPE1) of HCC patients.

Considering all these observations, we checked whether sAPE1 may serve as a biomarker for HCC. This work was undertaken to answer this issue and could represent a further step in biomarker discovery associated to patient’s prognosis, helpful to ameliorate therapy efficacy. Also, we show here that extracellular APE1 may contribute to triggering a pro-inflammatory state being able to promote IL-8 expression in a hepatic cancer cell line. Our novel findings open new perspectives in HCC biomarker discovery as well as APE1 functional role in cancer development.

## RESULTS

### Baseline characteristics

The demographic features of the groups are shown in Table 1. The participants were predominantly male (78%, 62%, and 71%) with a median age of 72, 67, and 56 years, respectively (*p* < 0.001). The etiology of chronic liver disease was alcohol abuse and/or metabolic in the majority of HCC and cirrhotic patients (55% and 40%, respectively), while viral hepatitis was in 23% and 16%.

**Table 1:** Demographic characteristics of the study populations

	HCC	Cirrhosis	Healthy	*P* value
**Patients characteristics**				
**Age (mean, 95%CI)**	71 (68.8–72.1)	66 (63.6–68.3)	56 (55.1–56.6)	<0.001
**Sex (M/F)**	77/22	31/19	71/29	ns
**Etiology**				ns
Alcohol metabolic	54	20		
Alcohol metabolic viral	18	17		
Viral	23	8		
Other^*^	4	5		
**Disease scores**				
CTP A/B/C	73/20/3	40/7/3		ns
BCLC 0/A/B/C–D	7/58/26/6			
ECOG 0/1/2–3	84/7/5			
GRETCH Low/Intermediate/High	15/67/1			
CLIP 0/1/2/3–4	27/32/16/9			
ITA.LI.CA Prognostic 2/3/4/5–8	44/21/12/7			
**Tumor parameters**				
**Number of lesions**				
Single <2 cm	15			
Single or 3 <3 cm	46			
Large-single or multi	37			
**Alpha fetoprotein**				
<20 ng/mL	52			
20–400 ng/mL	13			
>400 ng/mL	12			

^*^autoimmune, hemochromatosis, cryptogenic, usually in combination with other.

As of cirrhosis severity, most of the patients were CTP score A for 74% and 80%, whereas it was B or C in 23% and 20% for HCC and cirrhosis group, respectively. No significant difference was noticed between the two groups.

In HCC group BCLC 0, A, B, C/D scores were recorded in 7%, 59%, 26%, and 7% of patients, respectively. Regarding the prognosis scores, GRETCH score was intermediate in most patients (68%), low in 15%, and high in only 1%. CLIP score distribution appears to be more homogeneous, with a score 0 in 27% of the patients, score 1 in 32%, score 2 in 16%, and score 3/4 in 9%. As regards ITA.LI.CA Prognostic, score 2 was in 44%, score 3 in 21%, score 4 in 12%, and score >5 in 7%. ECOG score was 0 in the vast majority of HCC patients (85%), score 1 in 7%, and score 2/3 in 5% of the study group. No statistical differences exist among all groups.

In HCC group, tumor mass, number of lesions, and the level of alpha-fetoprotein (AFP) were recorded. With respect to tumor mass and number of lesions, 15% subjects showed a single nodule smaller or equal to 2 cm in diameter, 47% had either a single nodule ranging 2 to 5 cm, or 2–3 nodules up to 3 cm each, while 37% had single nodule larger than 5 cm in diameter or multifocal. As for AFP level, 53% had AFP level lower than 20 ng/mL, 13% between 20 ng/mL and 400 ng/mL, and 12% with AFP level above 400 ng/mL.

### Serum APE1 level significantly distinguishes HCC from cirrhotic patients and healthy subjects

The level of sAPE1 was measured by ELISA and expressed as median (95% CI), as previously described [16]. As a validation of the specificity of the ELISA assays used, we included a pool of blood donors’ sera spiked with different concentrations of recombinant purified APE1 protein (data not shown).

**Figure 1:**
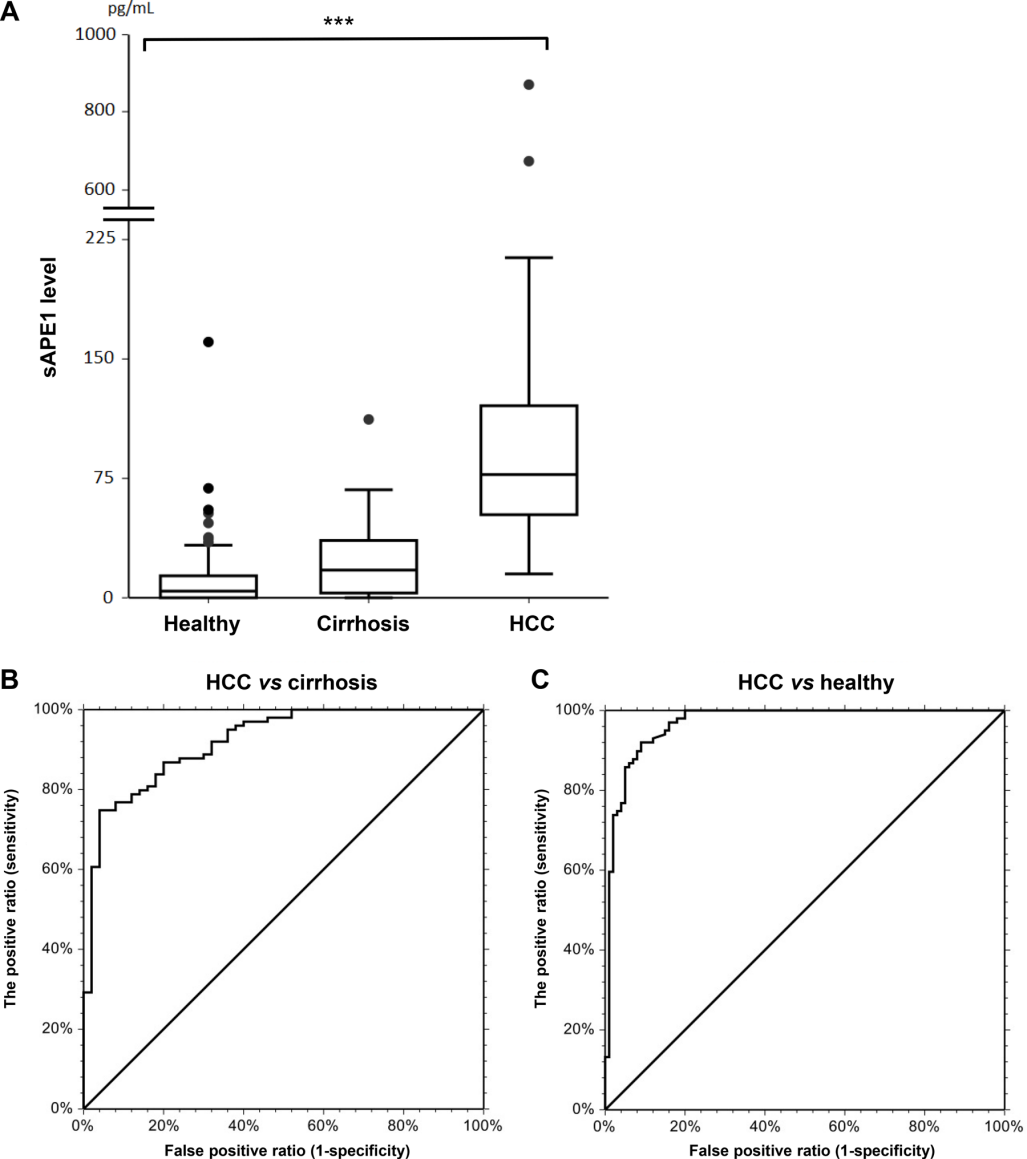
sAPE1 level in circulation (**A**) circulating sAPE levels in healthy blood donors, cirrhosis and HCC groups. ^***^P < 0.001 among groups. (**B**) ROC curve for HCC diagnostic from cirrhosis with AUC score of 0.87 (0.78–0.92, 95%CI) with 36.3 pg/mL as a cut-off. (**C**) ROC curve for HCC diagnostic from healthy blood donors with AUC score of 0.98 (0.96–0.99, 95%CI) with 19.0 pg/mL as a cut-off.

The highest concentration of sAPE1 was found in HCC, with median concentration of 75.8 (67.3–87.9) pg/mL with a minimum and maximum value of 15.2 and 881.4 pg/mL, respectively, and it was significantly higher (*p* < 0.001) compared to median concentration in either cirrhosis (29.8; 18.3–36.5 pg/mL) or healthy blood donors of (10.8; 7.5–13.2) (Figure 1A). sAPE1 was undetectable in 36 (36%) of healthy and in 10 (20%) of cirrhotic samples. A significant difference was observed also in cirrhosis compared to healthy control (*p* < 0.001).

### Circulating sAPE1 as diagnostic biomarker for HCC

When considering the diagnostic value of sAPE1, the area under the curve (AUC) of the receiver operator characteristic (ROC) curve was 0.87 (0.78–0.92) (Figure 1B). Using a cut-off of 36.3 pg/mL, sAPE1 can distinguish HCC patients from cirrhotic subjects with sensitivity and specificity of 88% and 71%, respectively. When considering the predictive value of sAPE1 in distinguishing HCC patients from healthy subjects, the AUC score improves to 0.98 (0.96–0.99) (Figure 1C). At a cut-off determined at 19.0 pg/mL, the sensibility and specificity increased to 98% and 83%, respectively.

### sAPE1 level in HCC is associated with viral infection but not with other clinical parameters

sAPE1 levels were significantly higher when comparing HCC-related viral infection to other non-viral etiologies (*p* < 0.001). In patients with either viral hepatitis C or B infection, the median sAPE1 was 98.8 (85.4–141.1). However, highest sAPE1 level was observed in patients with multiple etiologies (viral, metabolic, and alcohol) with median concentration of 132.37 (84.3–208.2).

The association of sAPE1 level and clinical and pathological characteristics of HCC patients was shown in Table 2. With respect to HCC staging and prognostic scores, there was a significant correlation between sAPE1 and GRETCH score, but not with any other HCC scores. No correlation with other clinical parameters, such as AFP, transaminases, transferrin, albumin, and insulin was observed.

**Table 2:** sAPE1 level and its association with clinical and pathological characteristics of the HCC group

sAPE1 (pg/mL)			
	Count	Median (95% CI)	*p* value
**Age**			ns
<72	52	74.2 (60.5–96.4)	
>72	47	78.8 (66.2–103.7)	
**Sex**			ns
Male	77	73.9 (63.2–87.2)	
Female	22	97.1 (65.0–122.9)	
**Etiology**			<0.001
Alcohol metabolic	54	62.6 (48.6–73.9)	
Alcohol metabolic viral	18	132.37 (84.3–208.2)	
Viral	23	98.8 (85.4–141.1)	
Other^*^	4	63.2	
**CTP**			ns
A	73	78.8 (65.0–96.4)	
B/C	23	70.76 (44.5–128.6)	
**BCLC**			ns
0-A	65	77.3 (60.5–96.4)	
B-C-D	32	74.5 (48.6–101.3)	
**GRETCH**			<0.05
Low	15	60.5 (33.0–101.3)	
Intermediate-high	68	83.2 (68.1–91.1)	
**CLIP**			ns
0–1	59	78.7 (67.3–97.7)	
2–3–4	25	71.6 (56.1–114.7)	
**ITA.LI.CA prognostic**			ns
2	44	87.1 (69.7–107.8)	
3	21	74.6 (56.1–108.7)	
4	12	53.5 (32.3–159.2)	
5–8	7	71.6 (40.3–138.3)	
**Number of lesions**			ns
Single <2 cm	15	86.9 (43.8–120.4)	
Single or 3 <3 cm	46	74.6 (60.5–117.9)	
Large-single or multi	37	74.5 (61.2–89.5)	
**AFP**			ns
<20 ng/mL	52	76.7 (65–87.2)	
20–400 ng/mL	13	138.3 (37.1–179.9)	
>400 ng/mL	12	80.5 (43.8–122.9)	

### APE1 expression is higher in HCC compared to its surrounding and normal tissue

**Figure 2:**
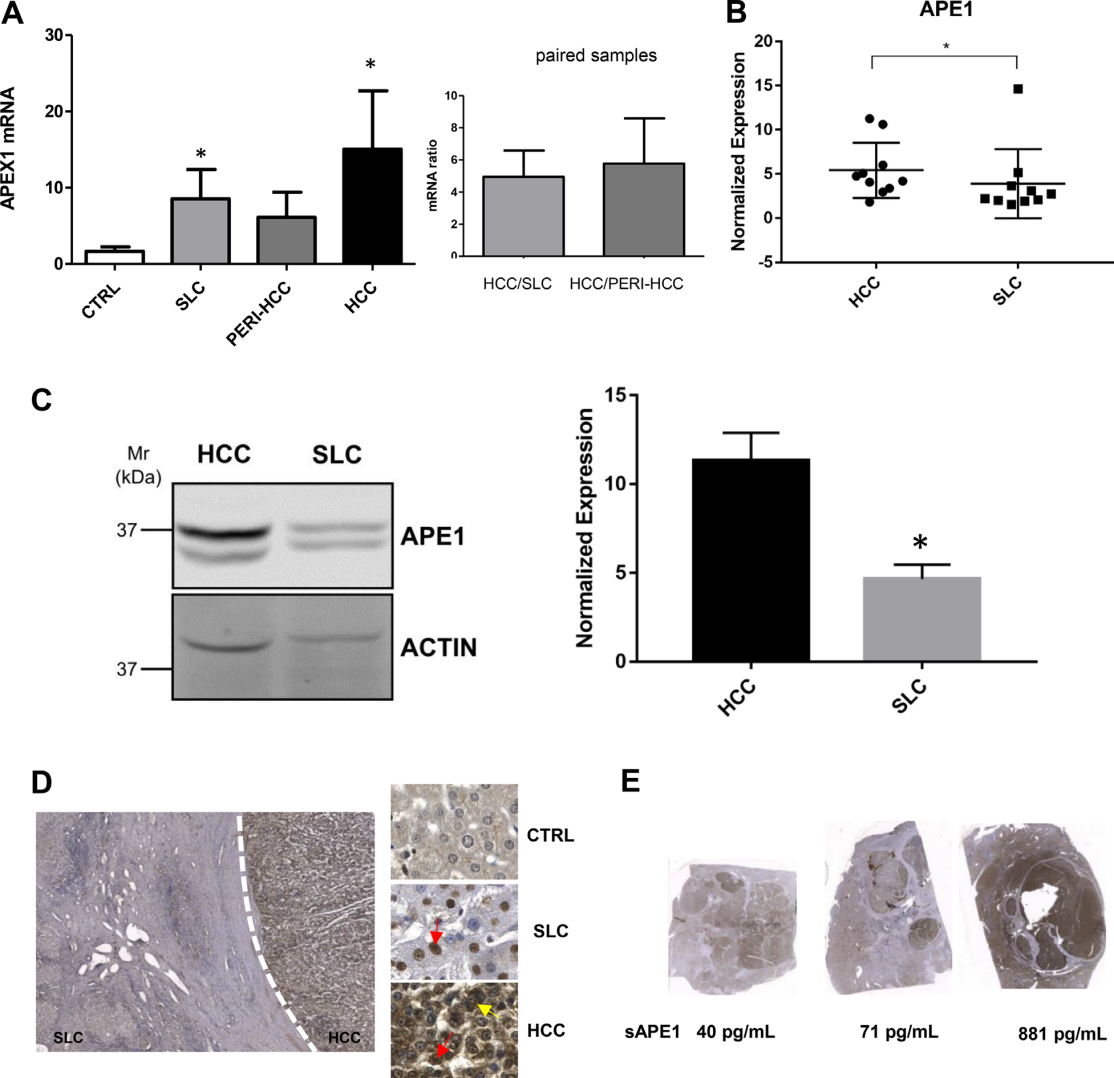
APE1 expression in HCC tissues (**A**) qPCR of tumor (HCC), peri-HCC, and surrounding liver cirrhosis (SLC), and normal liver (CTRL) (left panel). Ratio between HCC and SLC and HCC and peri-HCC within the same patients (right panel). APE1 mRNA quantification was normalized to two reference genes 18srRNA and Actin. Bar graphs indicate mean and SEM. (**B**) APE1 protein quantification in HCC and SLC tissue lysates from HCC cancer patients. Graphs indicate the different distributions of the fold of protein expression for each sample as the ratio between APE1 and actin. (see Supplementary Figure 1). ^*^P < 0.05. (**C**) Western blot analysis of HCC and SLC tissue lysates patients performed on pooled samples from HCC cancer. Actin was used as loading control and for the relative normalization. A representative image of western blot analysis is shown. Data represent the means of ± SD of three independent experiments. ^*^P < 0.05. (**D**) Immunohistochemistry of HCC, SLC, and normal (CTRL) tissue. Red and yellow arrows indicate nuclear and cytoplasmic positivity, respectively. (**E**) Scan of HCC nodules and its corresponding sAPE1 from 3 patients representing for each low, median, and high sAPE1.

In order to determine whether high sAPE1 levels found in HCC tumor samples correlate with an overall overexpression of APE1 both at protein and mRNA levels, Western blot analysis and qPCR were performed in non-tumor and tumor tissue of liver cancer. APE1 mRNA expression was analyzed in 59 tissues from 24 HCC patients, and 4 normal liver (CTRL). As shown in Figure 2A, an increase of APE1 mRNA was observed in HCC as compared either to the SLC and peri-HCC or also to normal liver (CTRL). When the mRNA expression was compared in paired samples of HCC to its corresponding SLC and peri-HCC, the ratio of mRNA up-regulation between HCC/SLC and HCC/peri-HCC was 4.9 ± 6.9 and 5.8 ± 11.3-fold, respectively.

APE1 protein levels were quantified in tissues from patients with high sAPE1 levels. Analysis were performed either within single patients (Figure 2B and Supplementary Figure 1) and pooled samples (Figure 2C). APE1 protein expression levels were determined by densitometric scanning of the immunoreactive bands and normalized against actin loading control. Besides the full-length protein band, a truncated form of the protein was detectable (Figure 2C), corresponding to a proteolytic form called NΔ33 (Supplementary Figure 2) [22]. A significant increase of APE1 total protein was observed in tumor samples, particularly in the full-length form (Figure 2C).

These results were confirmed by immunohistochemistry staining (Figure 2D), where APE1 protein resulted highly expressed in the HCC nodule compared to its paired SLC, while it was not noticed in normal liver. In SLC, the positivity of APE1 was mostly noticed in the nucleus, while in HCC tissues, both strong nuclear and cytoplasmic expressions were detected. To check whether the sAPE1 might be correlated with its expression in HCC nodule, the level of sAPE1 and hepatic APE1 protein were compared within the same patients. As shown in Figure 2E, the high sAPE1 expression corresponded with that found in HCC nodules.

### Recombinant exogenously added APE1 protein promotes the expression of IL-6 and IL-8 genes in JHH-6 cell line

**Figure 3:**
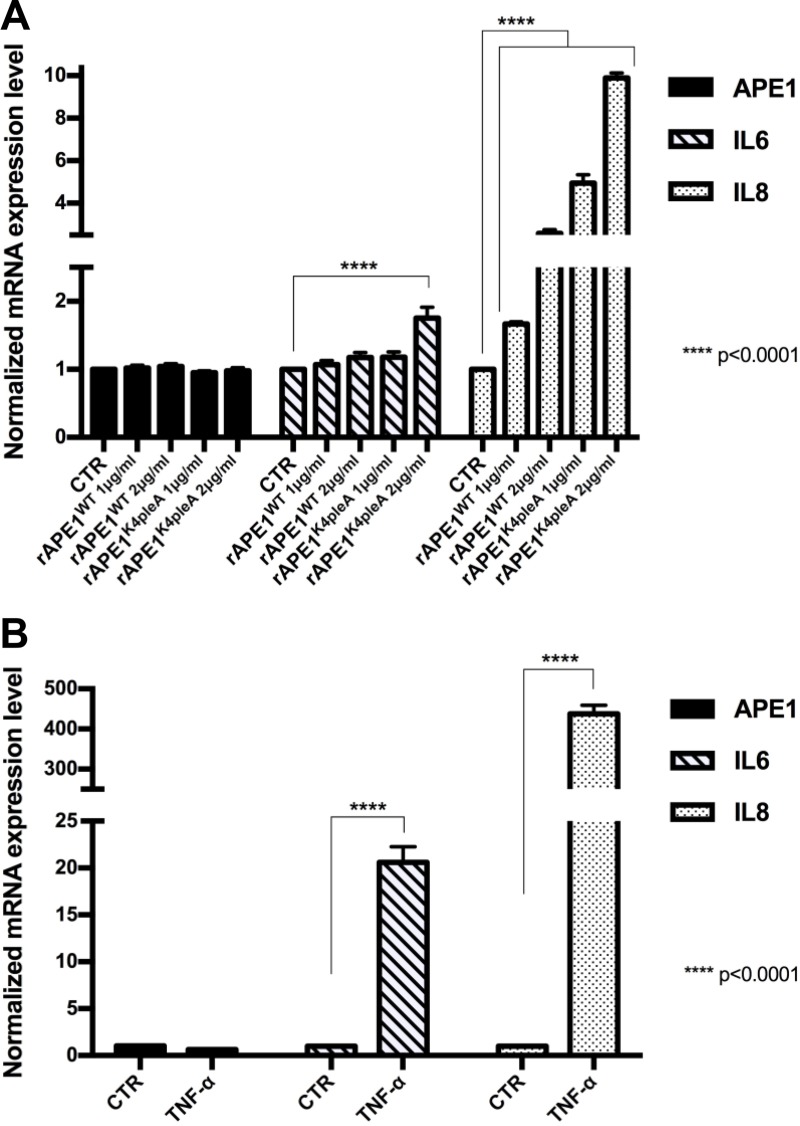
Recombinant APE1 promotes IL-6 and IL-8 mRNA expression in JHH-6 HCC cell line (**A**) JHH-6 cells were treated for 24 h in serum free medium with recombinant rAPE1^WT^ and rAPE1^K4pleA^ proteins (1–2 μg/ml concentration). 24 h serum free medium JHH-6 cells were used as a control (CTR). Performed qPCR analyses show that rAPE1^K4pleA^ recombinant protein promotes an induction of IL-6 and IL-8 mRNA expression levels. (**B**) IL-6 and IL-8 induction in JHH-6 TNF-α treated cells. JHH-6 cells were treated for 3 h in serum free medium with TNF-α (2000 U/ml concentration). Performed qPCR analyses show the induction of IL-6 and IL-8 mRNA expression mediated by TNF-α. Statistical analyses were achieved by using Two-way ANOVA test.

In order to evaluate a possible role for secreted APE1 in HCC, we tested whether exogenous addition of recombinant purified APE1 (rAPE1) may trigger a pro-inflammatory status in JHH-6 HCC cell line, as recently demonstrated in human monocytes cell lines [19]. In addition to the wild-type protein (rAPE1^WT^), we used an acetylated-mimicking mutant on residues 27, 31, 32, 35 (rAPE1^K4pleA^), in which Lys residues have been replaced by Ala, as described before [23, 24] and whose relevance in cancer have been recently demonstrated [22] (Supplementary Figure 3). As previously described, cells were treated for 24 hours with two doses of recombinant purified proteins, and the expression of IL-6 and IL-8 genes was assayed by qPCR. APE1 recombinant proteins induced an upregulation of both cytokines in treated cells; the rAPE1^K4pleA^ promoted a higher increase of IL-6/IL-8 compared to rAPE1^WT^ (Figure 3A). The cells responsiveness to a proinflammatory stimulation was verified by treating cells with rTNF-α (Figure 3B). This stimulatory activity of rAPE1 was also confirmed, even to different extents, in other cancer cell lines, such as: A549 from lung cancer, confirming the generality of this phenomenon that requires further additional careful characterization. However, we checked the stimulatory activity by rAPE1 using also GST-tagged rAPE1 as well as recombinant Glutathione –S-Transferase (a bacterial protein) alone obtained from the same *E. coli* strain. Data obtained (not shown) clearly demonstrated that, while GST-APE1 exerts the same stimulatory activity of rAPE1, the GST alone did not produce any effect on IL-6/-8 induction, eliminating the possibility of IL-6/-8 induction by any contaminating LPS or other proteins. These data suggest a role of sAPE1 as a paracrine pro-inflammatory molecule, which may modulate the inflammatory status in cancer microenvironment. However, in clinical samples there were not significant correlation between sAPE1 with both IL-6 and IL-8 levels in sera and in tissues (Supplementary Figures 4 and 5).

## DISCUSSION

In the present study, we found that sAPE1 protein, an essential DNA repair enzyme in the BER pathway, could be a promising diagnostic biomarker for HCC patients. Interestingly, this previously reported nuclear protein has been recently detected in the serum of patients of coronary artery disease [25] and bladder cancer patients where its level correlated with the level measured in cancer tissue [26]. More recently, this unexpected finding was confirmed in the plasma of both lipopolysaccharides-induced endotoxemic rats [27] and in NSCLC human patients [16], opening new perspectives in understanding the many functions of this unusual DNA repair protein in different conditions associated to oxidative stress and cancer development. We report that the sAPE1 levels correlate with its level in HCC tissue, in line with previous findings in non-small cell lung cancer [16].

The secretion of this protein seems to be a regulated phenomenon, depending on acetylation occurring on specific Lysine residues [17]. The biological relevance of secreted APE1 is still a matter of debate. It has been demonstrated that acetylated-APE1 may trigger apoptosis in TNBC cells by binding to the receptor for advanced glycation end products (RAGE) [28]. Other recent data demonstrated that the redox-function of APE1 may contribute to the control of the inflammatory response by inhibiting the TNF-α-induced endothelial inflammation via thiol-disulfide exchange in TNFR [18]. Moreover, very recently, a role for extracellular APE1 in the control of early stages of inflammation processes has been proposed [19]. Treatment of human THP-1 and RAW264.7 monocytes with rAPE1 increased the expression and secretion of the pro-inflammatory cytokine IL-6, through the involvement of NF-κB transcriptional activation, eliciting an autocrine/paracrine cellular response in a functional feedforward loop between APE1 and IL-6 regulation. However, detailed information regarding the mechanism responsible of APE1 release is still controversial, though it seems plausible that it might occur through extracellular vesicles formation via endosomal sorting complex [19].

Therefore, as a mainly nuclear protein, the source of sAPE1, whether it be from normal or cancer cells, is currently unknown and it is the focus of ongoing investigations. In our present study, we show a direct correlation between serum and tissue APE1 levels in the cohort we analyzed. We already described that the cytoplasmic expression of the protein in HCC patients correlates with poor prognosis, but we could not provide a significant biological role for cytoplasmic APE1 in HCC [20]. APE1 is a non-canonical secretory protein lacking a classic secretory signal based on protein sequence analysis. Intriguingly, several reports showed that APE1 is secreted under specific stimuli, such as those provided by Trichostatin-A [17] and LPS [19, 27]. According to these studies, secretion of APE1 was associated with its cytoplasmic translocation; however, in the present study, we failed to find a positive correlation between sAPE1 and its cytoplasmic distribution in HCC. Future work is warranted to inspect the secretory pathway of APE1 and its biological relevance. We believe that a passive release of sAPE1 by a necrotic process is unlikely, as its level does not correlate with any of the markers of liver damage analyzed. Moreover, cultured JHH-6 liver cancer cell lines do actively secrete APE1 in the culture media (data not shown) supporting the hypothesis that sAPE1 derives from an active secretion process involving, for instance, an exosomal pathway. Work is ongoing in our lab along this line to test this hypothesis.

Regarding the biological relevance of APE1 secretion, we found that recombinant exogenously added rAPE1 is able to promote IL-6/8 gene expression in JHH-6 HCC cell line. Two of the major pro-inflammatory cytokines secreted by senescent cells, IL-6 and IL-8, may function either to reinforce senescence [29] or to promote tumor invasiveness [30]. Thereafter, regulation of the inflammatory environment may have a critical role in determining the fate of both senescent and proliferating tumor cells. Based on the evidence that exogenous rAPE1 may trigger expression of both IL-6/8 cytokines, we may hypothesize that secreted APE1 may act as a paracrine molecule in regulating tumor microenvironment cell decision fates. These findings provide new insight into the underlying the clinical and biological relevance of circulating sAPE1, however, future work is needed to address these issues, which may have a strong relevance in the clinics of HCC prognosis and therapy.

## MATERIALS AND METHODS

### Patients

This case-control study was conducted in patients referring to the Liver Center of the University Hospital of Trieste, Italy. Fasting blood samples were collected in three groups: 99 consecutive patients patients observed between 2008 and 2018 whose HCC was diagnosed according to the EASL criteria [31]; 50 consecutive patients with cirrhosis confirmed by imaging, elastography, blood tests, and/or histological evaluation, without echographic evidence of HCC observed between April and June 2018; 100 consecutive healthy blood donors recruited in 2018 at the Transfusion Clinic.

Cirrhosis and HCC groups were further defined according to Child-Turcotte-Pugh score (CTP) and etiology (i.e. viral, alcoholic, metabolic, and other uncommon etiologies). For HCC group, classifications of Barcelona Clinic Liver Cancer (BCLC) [31], Eastern Cooperative Oncology Group (ECOG) [32], GRoupe d’Etude et de Traitement du Carcinoma Hépatocellulaire (GRETCH) [33], Cancer of Liver Italian Program (CLIP) [34], and the Italian Liver Cancer (ITA.LI.CA) [35] prognostic were defined. HCC samples were collected at the time of diagnosis, before any oncological treatment.

Exclusion criteria for all groups were age lower than 18 years old, pregnancy, and other malignancies. All the patients provided written informed consent and patient anonymity has been preserved. Investigation was conducted according to the principles expressed in the Declaration of Helsinki. The study was approved by the regional ethical committee (Comitato Etico Regionale Unico del Friuli Venezia Giulia, Prot. No. 2018 Os-008-ASUITS, CINECA no. 2225).

### Serum samples collection and sAPE1 quantification

Serum samples were collected in Vacuette^®^ tubes, centrifuged at 3500 rpm for 10 min and stored at –80°C until use. Hemolyzed samples were discarded from the analysis. Hemoglobin in serum was assessed with Beckman Coulter^®^DU^®^730 spectrophotometer using the Harboe Direct method [36] with Allen correction [37]. The considered cut-off for serum was 0.040 g/L. sAPE1 levels were determined using Human APEX1 ELISA kit (Cusabio, Houston, USA). The optical density (OD) was detected with an EnSpire microplate reader (PerkinElmer, Waltham, USA), at a wavelength of 450 nm with a correction set at 540 nm. sAPE1 levels (pg/mL) were then calculated by using a standard linear regression curve fitting by NCSS 11 Statistical Software (2016) (NCSS, LLC. Kaysville, USA). To determine the specificity of the test we spiked into blood donors’ serum 62.5 and 31.25 pg/mL of rAPEX1. We use the curve-fitting model built on OD vs sAPE1 concentration (pg/mL) to estimate the concentration of the two spiked samples (data not shown).

### HCC tissues collection and hepatic APE1 mRNA and protein expression

Fresh hepatic tissues were collected from HCC patients undergoing liver resection without any prior treatments. Samples consisted of HCC nodule, peri-HCC and surrounding liver cirrhosis (SLC). Tissues were quickly snap-frozen and stored in –80°C.

### Real time PCR

Total RNA was extracted using TriReagent (Sigma–Aldrich) and reversed transcribed using the iScript cDNA synthesis Kit (Bio-Rad Laboratories, Hercules, USA) according to the manufactures’ protocols. Reactions were run on IQ5 PCR detection system (Bio-Rad).

### Western blot

Lysates were resolved on 12% SDS-PAGE, transferred onto nitrocellulose membranes (Amersham^™^ Protran^™^, GE Healthcare) and probed with antibodies for APE1 (NB 100-116, Novus Biologicals, USA) (1:1000), APE1 N-terminal 1-14 aa specific goat polyclonal Antibody (NB100-897, Novus Biologicals) (1:2000) and actin (A2066, Sigma–Aldrich) (1:2000) as internal control. The IR-Dye labelled secondary antibodies (anti-rabbit IgG IRDye 680 and anti-mouse IgG IRDye 800) were used. Detection and quantification was performed with the Odyssey CLx Infrared imaging system (LI-COR GmbH, Germany). The membranes were scanned in two different channels using an Odyssey IR imager; protein bands were quantified using Odyssey software (Image Studio 5.0) and the relative signal was calculated.

### Immunohistochemistry

Paraffined hepatic slices were de-paraffinized with xylene and rehydrated with gradual concentration of ethanol. Tissues were incubated with APE1 antibody (NB100-101, Novus Biologicals, USA) followed by incubation with primary universal antibody enhancer and HRP polymer (Thermo Fisher Scientific, Cheshire, UK). The complex was visualized with the DBA peroxidase substrate kit (Vector Laboratories, UK) and nucleus was stained with hematoxylin. Slides were scanned by using Zenit G Sight 2.0 (A.Menarini Diagnostics, Italy) and images were generated using two microscopical systems Leica DM2000 (Leica, Wetzlar, Germany) and Zenit G Sight 2.0.

### Cell culture and treatments

JHH-6, an undifferentiated HCC cell line, were grown in William’s medium E (Sigma–Aldrich), supplemented with 10% fetal bovine serum, 100 U/ml penicillin, 100 μg/ml streptomycin (Euroclone, Milan, Italy). A total of 1.5 × 10^5^ JHH-6 cells were seeded in six-well plate for 24 h. For rAPE1 treatment, cells were treated for 24 h with rAPE1^WT^ or rAPE1^K4pleA^ with concentrations 1 μg/mL and 2 μg/mL in serum-free medium. While for TNF-α treatment, cells were treated with recombinant human TNF-α (Peprotech Inc., Rocky Hill, NJ) in serum-free medium using the same condition reported in [38].

Total RNA was extracted from JHH-6 treated cells using NucleoSpin^®^ RNA kit (Machery Nagel, Düren, Germany) according to the manufacturer’s instructions and reverse transcribed using SensiFAST cDNA Synthesis Kit (Bioline GmbH, Luckenwalde, Germany). qRT-PCR was performed with CFX96 Real-Time System, using SensiFAST™ SYBR^®^ No-ROX Kit (Bioline GmbH, Luckenwalde, Germany). APEX, IL6 and IL8 mRNA expressions were normalized to GAPDH and HPRT expression. The primers used were included in Supplementary Table 1.

### Recombinant APE1 protein expression and FPLC purification

Expression and purification of rAPE1 proteins from E. coli were performed as previously described [24, 39]. Briefly, E. coli BL21(DE3) cells were transformed with the construct pGex2T-APE1, coding for the glutathione S-transferase (GST)-APE1 full-length protein and for the APE1 acetylated mutant (K27A, K31A, K32A, K35A) and induced for 4 hours with 1.0 mM IPTG (Sigma–Aldrich) and collected upon centrifugation. The pellet was lysed and sonicated in the presence of protease inhibitor 2.1 mg/mL and Lysozyme 0.3 mg/mL. Samples were centrifuged at 23000 g for 20 minutes at 4°C, the supernatants were filtered and FPLC purified.

GST-tagged proteins were purified through a GSTrap column (GE Healthcare) and they were eluted in an increasing range of GSH concentration following the manufacturer’s instructions. The proteins were incubated with Factor X (Amersham) to remove the tag and separated from the enzyme through a benzamidine column (GE Healthcare). A cation exchange purification was performed. The fractions were pooled together and stored in buffer containing 25 mM Tris pH 7.5, 100 mM NaCl, 1 mM DTT, and 10% glycerol.

### Statistical analysis

Serum APE1 differences in a pairwise comparison were calculated by Mann–Whitney U. Differences among groups were calculated by Kruskal-Wallis test in one way ANOVA, with Bonferroni correction. The receiver operating characteristic (ROC) curves were plotted to estimate the diagnostic value of sAPE1. Analyses were performed by using NCSS 11 Software (2016) (NCSS, LLC. Kaysville, Utah, USA, https://www.ncss.com/software/ncss/).

## SUPPLEMENTARY MATERIALS FIGURES



## Abbreviations

AFP: Alpha-fetoprotein
APE1: Apurinic/apyrimidinic endonuclease 1
AUC: Area under the curve
BCLC: Barcelona Clinic Liver Cancer
BER: Base excision DNA repair
CLIP: Cancer of Liver Italian Program
CTP score: Child-Turcotte-Pugh score
CTRL: Control
DDR: DNA damage responses
ECOG: Eastern Cooperative Oncology Group
GRETCH: GRoupe d’Etude et de Traitement du Carcinoma Hépatocellulaire
HCC: Hepatocellular Carcinoma
ITA.LI.CA: Italian Liver Cancer
NSCLC: Non-Small Cell Lung Cancer
OD: Optical density
rAPE1: recombinant apurinic/apyrimidinic endonuclease 1
rAPE1^K4pleA^: acetylated-mimicking mutant recombinant APE1 on residues 27, 31, 32, 35
rAPE1^WT^: recombinant wild-type APE1 protein
ROC curves: receiver operating characteristic curves
sAPE1: Serum apurinic/apyrimidinic endonuclease 1
SLC: surrounding liver cirrhosis
