# Supplementary material for: Serum AP-endonuclease 1 (sAPE1) as novel biomarker for hepatocellular carcinoma
Source: Oncotarget. 2019 Jan 8;10(3):383–94. doi: 10.18632/oncotarget.26555 (PMC6349448; doi:10.18632/oncotarget.26555)
Supplement: Supplementary file 1 [file oncotarget-10-383-s001.pdf]

Serum AP-endonuclease 1 (sAPE1) as novel biomarker for hepatocellular carcinoma

SUPPLEMENTARY MATERIALS

Supplementary Table 1: Primer list

gene	Forward primer	Reverse primer
18S RNA	5′- TAACCCGTTGAACCCCATT-3′	5′-CCATCCAATCGGTAGTAGCG-3′
ACTB	5′-CGCCGCCAGCTCACCATG-3′	5′-CACGATGGAGGGGAAGACGG-3′
APEX	5′-CTGCCTGGACTCTCTCATCAATAC-3′	5′-CCTCATCGCCTATGCCGTAAG-3′
GAPDH	5′ TCTCTGCTCCTCCTGTTC-3′	5′-GCCCAATACGACCAAATCC-3′
HPRT	5′-AGACTTTGCTTTCCTTGGTCAGG-3′	5′-GTCTGGCTTATATCCAACACTTCG-3′
IL-6	5′-CAAAGATGTAGCCGCCC-3′	5′-GTTCAGGTTGTTTTCTGCC-3′
IL-8	5′-CTGGCCGTGGTCCTCTTG-3′	′-CCTTGGCAAAACTGCACCTT-3′

Serum IL-6 and IL-8 quantification

IL-6 and IL-8 were quantified in 21 HCC patients using the Bio-Plex (BIO-RAD Laboratories, Milan, Italy) and array reader (Luminex, Austin, TX). In brief, 50 µL of serum and standard were added in a 96 multi-well plate containing analyte beads based on a magnetic bead multiplex immunoassays. After incubation for 30 minutes at room temperature and washing, the antibody-biotin reporter was added and incubated for 10 minutes with streptavidin-phycoerythrin. The Bio-Plex Manager software optimized the standard curves automatically and returned the data as Median Fluorescence Intensity (MFI) and concentration (pg/ml). This assay has a reported limit of detection of 1–20 pg/ml, depending on the cytokine target. Each sample was run in a blinded fashion, and the value of all individual data was collected for the analysis.

SUPPLEMENTARY RESULTS

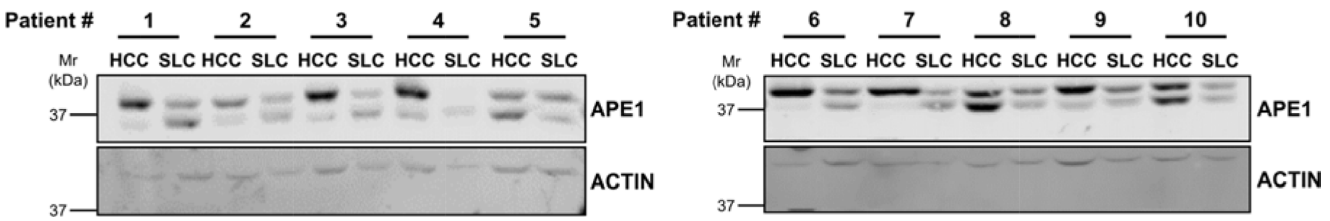

Supplementary Figure 1: APE1 western blot analysis performed on total extract of liver biopsy. Western blot analysis of tumor (HCC) and surrounding liver cirrhosis (SLC) tissue lysates from HCC cancer patients. Actin was used as loading control.

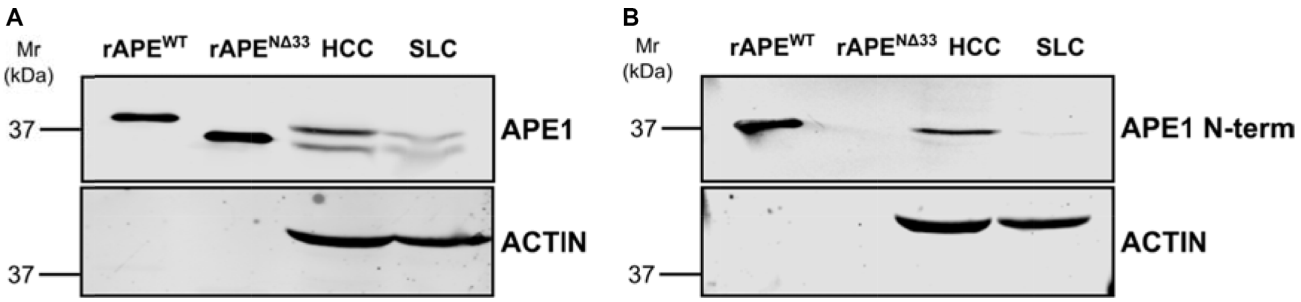

Supplementary Figure 2: The truncated form of APE1, present in tumor and non-tumor sample of HCC, is the N-terminal deleted protein corresponding to the NΔ33 form. Western blot analysis of tumor (HCC) and surrounding liver cirrhosis (SLC) tissue lysate performed on pooled sample from HCC cancer patients with monoclonal antibody for APE1 (**A**) or with an N-terminal-specific antibody of APE1 (**B**). Actin was used as loading control and a full-length recombinant purified protein of APE1 ($rAPE1^{WT}$) and a 33-N-terminal truncated form ($rAPE1^{N\Delta33}$) were used as molecular-weight size marker.

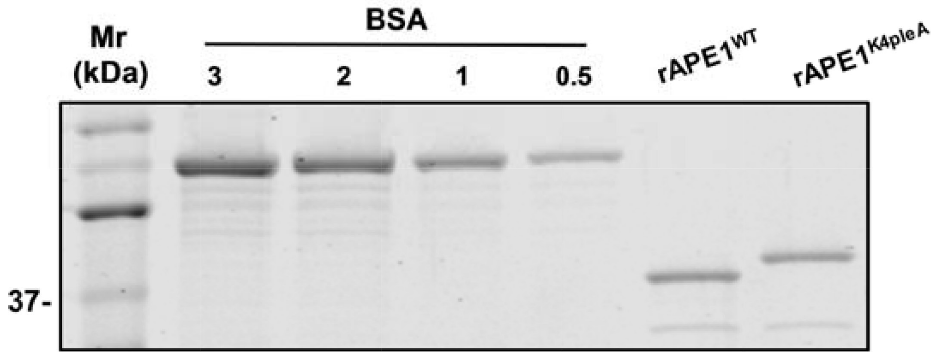

Supplementary Figure 3: Recombinant APE1 proteins quantification. Comassie brillant blue staining of $rAPE1^{WT}$ and $rAPE1^{K4pleA}$ (1 µl loaded). A standard curve was carried out, loading respectively 3, 2, 1, 0.5 µg of BSA and it was used as a reference to perform the quantification of recombinant APE1 proteins.

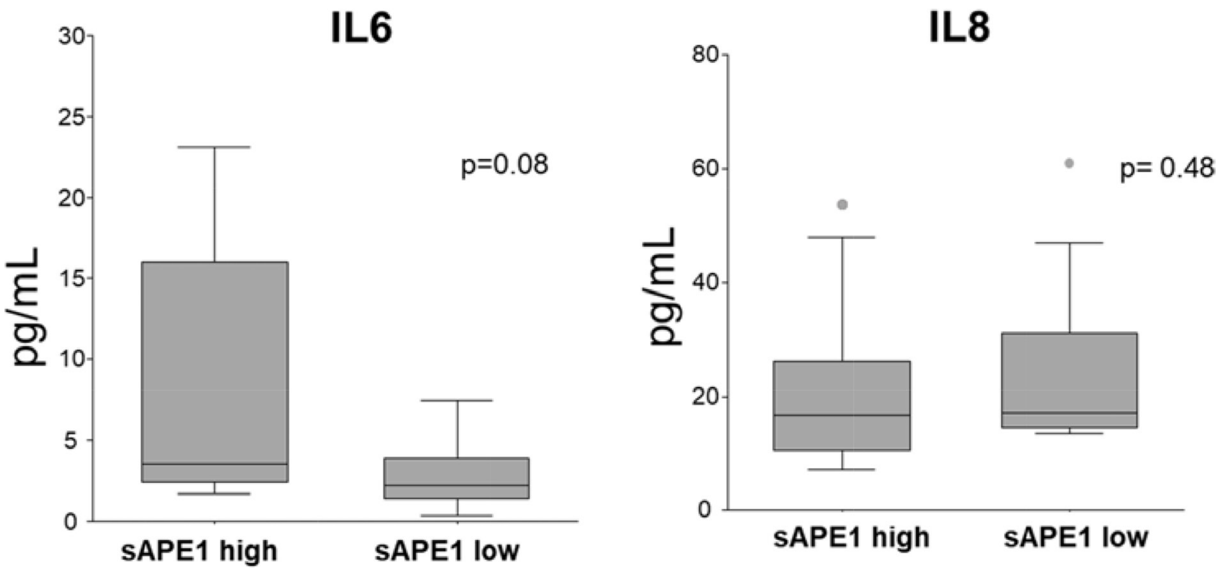

Supplementary Figure 4: Serum IL-6 and IL-8 in HCC patients. We assessed the concentration of both IL-6 and IL-8 in sera of 21 HCC individual samples included in the APE1 cohort. Patients were grouped based on median sAPE1 level: sAPE1 high (>75.8 pg/mL, n = 11) and sAPE1 low (<75.8 pg/mL, n = 10). The level of IL-6 was slightly higher in sAPE1 high group compared to sAPE1 low (median values 3.16 and 2.18 pg/mL, respectively) even though it was not significant (p = 0.08). No differences were observed between the IL-8 groups (median values 16.8 and 17.12 pg/mL, for sAPE1 high and sAPE low, respectively). Based on this data, we presume that in the circulation, there is no significant correlation between sAPE1 and neither IL-6 and IL-8 levels. This was somehow expected, since inflammatory response is affected by multiple factors and not only by the tumor and the response of cancer cells to exogenous APE1 protein stimulation could be a transient phenomenon rather than a chronic mechanism of triggering inflammatory response.

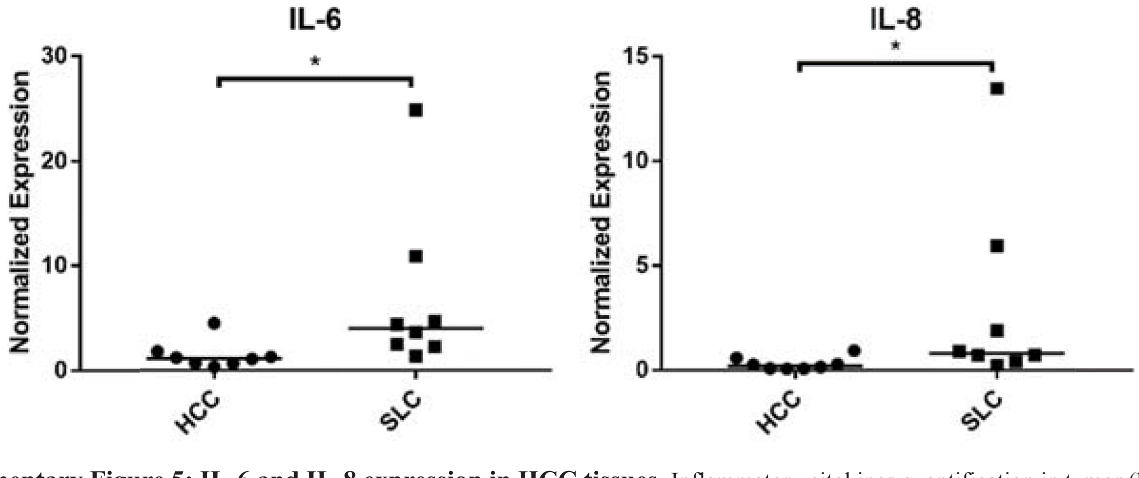

Supplementary Figure 5: IL-6 and IL-8 expression in HCC tissues. Inflammatory citokines quantification in tumor (HCC) and surrounding liver cirrhosis (SLC) tissue lysates from HCC cancer patients used in the Figure 2B of the Main MS. IL-6 and IL-8 mRNA quantification was normalized to two reference genes GAPDH and Actin. Bar graphs indicate median. Neither expression of IL-6 nor IL-8 seem to be correlated with tissue levels of APE1 protein. Again, the lack of an apparent strong correlation between the tissue levels of IL-6 and IL-8 with sAPE1 can be explained on the basis of a chronic inflammatory condition occurring in the tissue cancers which is not recapitulated in the cancer cell lines model, that, on the contrary, represent an acute condition of stimulation with extracellular recombinant APE1 protein.
